# Immunology Education Without Borders

**DOI:** 10.3389/fimmu.2019.02012

**Published:** 2019-08-28

**Authors:** Dieter Kabelitz, Michelle Letarte, Clive M. Gray

**Affiliations:** ^1^Institute of Immunology, University of Kiel, University Hospital Schleswig-Holstein Campus Kiel, Kiel, Germany; ^2^Department of Immunology, The Hospital for Sick Children, University of Toronto, Toronto, ON, Canada; ^3^Division of Immunology, Institute of Infectious Diseases and Molecular Medicine, Groote Schuur Hospital, University of Cape Town, Cape Town, South Africa

**Keywords:** Education Committee, immunology courses, Immunopaedia, IUIS, on-line learning

## Abstract

One of the mandates of the International Union of Immunological Societies (IUIS) is to promote immunological education to young scientists across the globe, including a large focus on those from low and low-to-middle income countries (LIC and LMIC). It strives to achieve this goal through the Education Committee (EDU), which is one of ten committees of the IUIS. To this end, EDU organizes three to four one-week courses per year in close cooperation with regional immunological societies and local organizers. Initially, the focus has been on Africa, addressing the most relevant topics and health issues facing specific countries or regions in the continent. The idea was then extended to Latin America and now also includes courses in Asia. The faculty of all courses is a blend of international and local/regional experts also known for their teaching expertise. The courses are highly interactive, and include “meet-the-speakers” sessions, poster walks, and sessions on grant or PhD project writing, and on practical aspects of becoming a successful scientist. Importantly, all the IUIS-EDU courses use a combination of pre- and during-course on-line learning followed by consolidation of knowledge in a collegial setting. This “flipped” classroom approach ensures that participants have acquired the basic knowledge needed to optimize their participation in the course. Immunopaedia is the IUIS-endorsed immunology learning site used for this purpose. All faculty members are requested to contribute material related to their specific topic while students must learn the on-line material before coming in person to the course. All course participants have free access to all Immunopaedia material indefinitely. The implementation of regional immunology courses targeted to local health issues in areas of the world where PhD students, post-doctoral, and early career scientists often do not have access to open on-line resources and contact with renowned experts in the field has proven to be highly successful. The long-term impact of this structured educational program is already visible through the large number of young scientists who are now connected via Immunopaedia and who are forming networks in regions where there had been very little contact before and building new Immunological Societies.

## Introduction

The International Union of Immunological Societies (IUIS) is the umbrella organization for regional federations composed of national societies of immunology throughout the world. There are four regional Federations (EFIS, European Federation of Immunological Societies; FAIS, Federation of African Societies of Immunological Societies; ALAI, Latin American Association of Immunology; FIMSA, Federation of Immunological Societies of Asia-Oceania) with currently 77 member societies and > 60,000 immunologists worldwide (http://www.iuisonline.org). While the overall goals of the IUIS are to promote cooperation and communication between member societies and to contribute to the advancement of immunology in all fields and aspects, most of the implementation of these goals are carried out by the ten committees: Clinical immunology, Education, Gender Equality and Career Development (GEC), Inborn Errors of Immunity, Immunotherapy, Nomenclature, Publication, Quality Assessment and Standardization, Vaccine, and Veterinary Immunology. The task of the Education Committee (EDU) is to promote immunological education and disseminate knowledge to students and young scientists in LICs and LMICs of the world. The EDU Committee is truly international and is currently comprised of 17 members from 16 different countries from around the world, representing the four regional federations and North America (US and Canada) (see Supplemental Table 1). In the following sections, we expand on the various activities of the EDU Committee in recent years, including the sponsoring of new courses in LIC/LMICs and providing travel awards for young immunologists from these regions to attend specific well-established training courses. We then focus on the organization of our own brand of immunology courses and describe Immunopaedia (https://www.immunopaedia.org.za/), which is the IUIS endorsed immunology learning site that supports IUIS-EDU courses in LIC/LMICs. For each course, a separate content composed of several modules is generated where relevant topic-related teaching material (reviews, lectures, background information) is collected and made freely available to students. All course participants have free access to the content, not only pertaining to their specific course, but to a rich spectrum of immunology material related to all courses and for an indefinite period.

## Activities of the EDU Committee

The EDU Committee acknowledges the benefit that young scientists from LIC/LMIC experience when participating in high-level training courses abroad. In cooperation with the American Association of Immunologists (AAI), EDU has selected each year since 2008, three (and now four) students to attend each of the AAI Introductory and Advanced Immunology Courses. In partnership with the Federation of Clinical Immunology Societies (FOCIS), we have also awarded three fellowships per year between 2009 and 2016, for students to participate in the FOCIS Advanced Course in Basic & Clinical Immunology. The IUIS Gender Equality and Career Development Committee (GEC) has helped us financially by sponsoring one student for each AAI course per year and since 2017 has taken over the selection and co-sponsoring of the FOCIS awards. In collaboration with the German Society for Immunology (DGfI), EDU supports three travel awards for candidates selected by DGfI together with those wishing to attend the Ettal Spring School (https://dgfi.org/akademie-fuer-immunologie/spring-school/; see Table 1).

**Table 1:** Activities of the IUIS-Education Committee.

**A. Ongoing annual activities:** Since 2008, in collaboration with the American Association of Immunologists (AAI) we have selected annually 3 (now 4) students from the LIC/LIMCs and co-sponsored their participation in the AAI Introductory and Advanced Immunology Courses, respectively. The IUIS-GEC committee provides one of the travel awards Since 2011, we have supported annually 3 travel fellowships for students from LIC/LMICs and East-European countries to attend the Ettal Spring School of the German Society for ImmunologyB. **B. Previous activities:** 2009–2016, selection and support of 3 students to attend the FOCIS -Advanced Course in Basic and Clinical Immunology 2012–2017, support for the EFIS-EJI South Eastern European Immunology School (SEEIS), in Sarajevo, Bosnia and Herzegovina, Bulgaria, Romania, Montenegro, Albania, and Ukraine, respectively Funding for the EFIS-EJI Ruggero Ceppellini Advanced School of Immunology, Milan (2013, 2014); Advanced WHO/TDR Course on Immunology, Vaccinology, and Biotechnology, La Paz, Bolivia (2008), Lausanne (2010); Immunoparasitology Conference, Woods Hole, USA (2010) Funding for several FIMSA courses in Queensland, Australia; New Delhi, India; Singapore Awards for the Introductory Course in Immunology in Sudan, 2008; Flow Cytometry Schools in Morocco (2013, 2016) and South Africa (2013); Mediterranean Courses of Immunology in Morocco (2007) and Algeria (2010); the ICGEB Training Course in Global Infectious Disease Research 2013, Cape Town, RSA Support for John Humphreys Advanced Immunology Courses in Moscow (2007) and Havana (2012) Contribution to the International Courses for Clinical Immunology of Infectious Diseases at Suez Canal University (2007, 2008) and the E-training Workshop on Mucosal Immunity/Vaccine in Fayoum University, Egypt, 2009 Travel awards for students to attend Regional Congresses (FIMSA Singapore 2015; FAIS Durban,RSA, 2012, Nairobi, Kenya 2014, Hammamet, Tunisia, 2017) and International Congresses (Rio de Janeiro, Brazil 2007, Kobe, Japan 2010) Funding of multiple ALAI courses: Immunomodulation 2009, Mucosal Immunology, 2010, Immunotherapies, 2010, in Buenos Aires, Argentina; First Argentinean Spring Course in Advanced Immunology, Los Cocos, 2013; Mucosal Immunology and Vaccination, Trinidad, Cuba, 2010; São Paulo Immunology Course, Brazil, 2011; First Meeting about Primary Immunodeficiencies in Latin America, Lima, Peru, 2011 **C. Unique activities:** Organized the BioLegend-IUIS Symposium on “Global Immunology Challenges for Young Investigators” at ICI 2016, Melbourne, Australia ISIA Advanced Course of Immunotherapy, Tehran, Iran, 2018

These travel fellowships are advertised on IUIS, FAIS, ALAI, FIMSA, AAI, and DGfI websites. For each of these courses, the number of applications by far exceeds the available fellowships, and it is a demanding task for the committees to make a fair candidate selection based on professional competence, gender balance, and region of origin. Depending on fund availability, EDU has contributed financially to several established courses by providing student travel awards. The EFIS-EJI South Eastern European Immunology School (SEEIS), organized annually in a different country, provides an update on immunology and practical exercises in technologies like flow cytometry or diagnostic autoantibody microscopy. EDU and GEC committees both provided travel awards to the Ceppelini EFIS-EJI Ruggero Ceppellini Advanced School of Immunology. Table 1 illustrates the many courses, workshops and international meetings to which EDU contributed financially since 2007.

Reports from the EDU supported activities can be found on the IUIS-EDU website (http://iuisonline.org/index.php?option=com_content&view=category&id=39&Itemid=81&5a48d87dcdf6fac96a6bc0ffc1b9e64c=6704e3a3cee3647430590629d6d2c3c4).

At the International Congress of Immunology 2016 in Melbourne, Australia, EDU obtained support from BioLegend to organize for the first time a symposium on “Global Immunology Challenges for Young Investigators.” BioLegend offered 4 travel awards that allowed the selected students to attend the main conference, share research findings and discuss their challenges with other international students and faculty.

Furthermore, EDU co-organized and co-sponsored (together with the IUIS Clinical Immunology Committee) the ISIA Advanced Course of Immunotherapy, Tehran/Iran in April 2018. Immunology has a long academic tradition in Iran, and this activity again underscores the ambition of the EDU Committee to support “Immunology Education without Borders.”

## The Concept of IUIS-EDU Courses

### Focus on Low and Low-to-Middle Income Countries

Immunology is among the fastest growing disciplines in contemporary biomedical research. Currently, we witness how immune modulating concepts and novel biologics modify and actually replace or complement established therapies at breathtaking pace. Introduction of immune checkpoint inhibitors has revolutionized cancer immunotherapy in recent years (as acknowledged by last year's Nobel Prize in Physiology or Medicine awarded to Drs. James P. Allison and Tasuku Honjo). Furthermore, the approval of cytokine-blocking biologics has helped enormously to ameliorate symptoms in chronic inflammatory diseases. The successful introduction of vaccines can still be considered the most effective preventive measure in medicine worldwide. Notably, however, efficacious vaccines are still in development and not yet available for some of the major infectious diseases (e.g., tuberculosis, malaria, schistosomiasis, HIV) which tend to predominate in LIC/LMICs worldwide. One such example is the outbreak of Ebola in West and Central Africa, reminding us of the urgent need for rapid responses and further research into basic and applied/clinical immunology in order to better understand the complexity of the immune system. This is important for the development of rational immune therapies in the same way this has been done for Ebola vaccination strategies. Given that the prevalence of such tropical and other infectious diseases is disproportionally high in less developed countries, it is mandatory to train the young PhD and clinician scientists in those parts of the world in such a way that they can become future scientific leaders. The ultimate goal is to have in-country immunology leaders who can attract funding and a critical mass of followers to offset the current poor infrastructure and facilities that are found in many LIC/LMICs. The mandate from the IUIS was to increase the numbers of regional courses using our unique brand of blending immunology knowledge with career skills building. This has meant networking and building teaching modules with local immunologists, identifying the most pressing health issues in the area and bringing international faculty to participate in an interactive course. Due to the fact that the EDU budget is very limited, fundraising then becomes an important task for each of the regional courses.

With this in mind, EDU has introduced the concept of “immunology plus,” where we aim to promote highly interactive courses that include informal meet-the-speaker sessions, poster presentations, grant writing training, PhD fellowship project writing, and practical sessions on how to prepare a CV and how to verbally interact with colleagues and faculty. The goal is to show students how to become a successful scientist through immunology. One key aspect of a thriving immunologist is the ability to network with peers and more established scientists. Thus, a critical aspect of every IUIS-EDU course is to allow all participants and faculty from around the world to interact and build lasting contacts. Efficient mentoring is most important for the positive establishment of an academic carrier, and we encourage both participants and faculty members to build up lasting mentorship relationships during the courses. We also put an emphasis on specifically encouraging women PhD students and researchers to enter a career in science, despite obvious obstacles in many places. Together with the Gender Equality and Career Development Committee (GEC) we organize sessions on women in science issues, with an inspiring and experienced guest who reflects on her personal career development and advises on gender-related issues. Dr. Olivera Finn, chair of GEC, and Dr. Narinder Mehra, GEC co-chair have organized and chaired several sessions at international meetings, including the FAIS 2014 meeting in Nairobi, Kenya (with guest Dr. Jane Kengeya-Kayondo, Wellcome Trust, Special Adviser for Africa); at ALAI 2015 in Medellin following our Immune-Columbia course (with guest Dr. Nancy Gore Saravia, Scientific Director and Research Leader for Leishmaniasis, CIDEIM); at the International immunology Congress in Melbourse, Australia, in 2016 (with guest Dr. Laurie Glimcher, President and CEO of Dana Farber Cancer Institute); at FAIS 2017 in Hammamet, Tunisia (with guest Dr. Oum Kalthoum Ben Hassine, Founder and Director of the Research Unit of Biology, Ecology and Parasitology of Aquatic Organisms at the University of Tunis); and at ALAI Meeting in Cancun, Mexico, in 2018 (with guest Dr. Clara Gorodezky, Head of the Department of Immunology and Immunogenetics of the Istituto de Diagnóstico y Referencia Epidemiológicos of the General Direction of Epidemiology at the Secretary of Health in Mexico). Dr. Michelle Letarte, past-chair of EDU, has managed several dinner discussion groups including at the Onco-Immunology Mexico Course in San Miguel de Allende in October 2017 and at the Immune-Ethiopia course in Gondar in March 2017. Dr. Miriam Merad, Mount Sinai Endowed Professor in Cancer Immunology, gave a scientific lecture as well as a career motivation dinner talk at the Morocco course in Fes, in 2018. At the Immuno-Informatics course in Mexico City in April 2019, Dr Selene Fernandez-Valverde, a young Mexican investigator with already an outstanding career at the intersection of bioinformatics and developmental evolutionary biology and who received L'Oreal-UNESCO Research Fellowship For Women in Science Award and International Rising Talents Distinction in 2016 and 2018 respectively, reflected on her personal career development and how it was modulated by family and other gender-based issues. Such exemplary role models can help to motivate young women PhDs to pursue a career in science and to ensure and reassure them that they are not alone.

### Application and Selection Procedures

IUIS-EDU Courses are advertised on the websites of IUIS, its Federations and Regional Societies, Immunopaedia, and other relevant networks. Applicants are usually requested to provide a letter of motivation, a short CV (with a publication list, if applicable), a letter of support from their supervisor, and an abstract to be presented as a poster and/or short talk. All applications are reviewed by a scientific committee consisting of members of EDU, the Regional Federation and/or Society, and local scientists. Selection of candidates is based on the respective ranking and additional aspects such as gender balance and country of origin. Generally, students from ten or more countries attend the course and generate an enormous amount of energy and healthy competition. The faculty of all courses is a blend of international and local/regional scientists. Whenever possible, faculty and participants stay at the same hotel to foster informal interactions during breakfast and dinner. Importantly, all the EDU courses use a combination of on-line pre-learning with consolidation of that knowledge during contact time on the course. This “flipped” or blended classroom approach ensures that participants learn or review basic immunology modules on-line prior to contact time. Our adapted approach has been based on several active learning approaches in science, engineering and mathematics (1) as well as innovative teaching in immunology (2). Such an approach has allowed a more interactive and a more discursive 5–7 day course around the theme of the meeting. Evaluation of the course has so far been around the enjoyment and use of Immunopaedia and the ease of learning. Besides holding pre- and post-MCQs to assess short-term knowledge gain, being able to assess whether application and synthesis of immunology knowledge is gained has yet to be made and the IUIS would need to collaborate with immunology education specialists to achieve this goal. Despite this, Immunopaedia is the IUIS endorsed immunology learning site that is used for all our courses in LMICs (see below). The long-term impact of the structured educational program initiated by IUIS-EDU is already visible, e.g., through the formation of networks of young scientists in regions where there had been very little contact before. One example is WAYII, the West African Immunologists' platform launched in 2016 by Léonce Kouakanou and Ulrich Fabien Prodjinotho during the IUIS-FAIS-IMMUNO-GAMBIA course in Banjul/The Gambia. WAYII aims to promote Immunology in Africa by providing an interactive interface for network; to establish and/or reinforce regional collaborations; to enhance transfer of skills and knowledge, and finally to help in organizing meetings, trainings, conferences. More information on WAYII activities is available on the Facebook page: https://www.facebook.com/WestAfricanYoungImmunoInvestigators/. In cooperation with the Federation of African Immunological Societies (FAIS), we have initiated the “Africa Immunology School” concept where on average two courses per year are organized on the continent, rotating between the five regions of Africa. These courses usually accept 40 to 50 participants (PhD students, post-docs, young scientists, and medical doctors) from the host and surrounding countries, with topics adapted to the specific regional needs. In line with the large burden of disease on the Continent, a major topic of courses in Africa focus on the big three: HIV, tuberculosis, and malaria, although there are regional differences in the importance of these. Other significant themes have included Ebola, helminth infections, or infection-associated cancers. A second geographical focus of IUIS-EDU activities is Latin America where courses have been organized in Columbia, Brazil, and Mexico in collaboration with the Latin American Association of Immunology (ALAI) on topics ranging from Vaccines to Immunoregulation and Bioinformatics. Currently we are extending our activities to additional regions: a first course organized in collaboration with the Federation of Immunological Societies of Asia-Oceania (FIMSA) will take place in Jaipur, India. Since the inception of this program in 2015, 11 courses have been organized (Colombia, South Africa [x2], Tunisia, Mexico [x2], Ethiopia, Gambia, Brazil, Morocco, Kenya) and 6 more are being planned (South Africa, India, Benin, Ethiopia, Algeria, Argentina). An overview of topics and locations of IUIS-EDU courses since 2015 is presented in Table 2.

**Table 2:** IUIS-Education Committee Courses since 2015.

**Year**	**Course**	**Topic**	**Dates**	**Location**
2015	IUIS-ALAI-IMMUNO-COLOMBIA	Immunoregulation in Health and Disease	October 10–13	Medellin, Colombia
2015	IUIS- IDA-SANTHE-FAIS- IMMUNO-SOUTH AFRICA 1	Biomarkers and Correlates of Immune Control of HIV, TB, and Malaria	October 20–24	Cape Town, RSA
2016	IUIS-FAIS IMMUNO-TUNISIA	Tolerance and Autoimmunity	April 4–8	Hammamet, Tunisia
2016	IUIS-ALAI-SMI ONCOIMMUNOLOGY-MEXICO	Oncoimmunology	October 5–8	San Miguel de Allende Guanajuato, Mexico
2016	IUIS-FAIS-IMMUNO-GAMBIA	Immunology of Infectious Diseases	November 19–26	Banjul, The Gambia
2017	IUIS-FAIS-IMMUNO-ETHIOPIA	New Developments in the Immunology, Diagnosis, and Treatment of Leishmaniasis, Schistosomiasis, and Helminth Infections	February 26–March 4	Gondar, Ethiopia
2017	IUIS-IDA-SANTHE-FAIS- IMMUNO-SOUTH AFRICA 2	Immune Tolerance and Evasion Strategies by Pathogens	September 1–6	Gordon Bay, RSA
2017	IUIS-ALAI-IMMUNO-BRAZIL	Advanced Course on Vaccines	December 8–11	São Paulo, Brazil
2018	IUIS-FAIS-SMI-IMMUNO-MOROCCO	Cancer, Inflammation, and Immunotherapy	April 3–7	Fes, Morocco
2018	IUIS-FAIS-IMMUNO-KENYA	How Viruses Hijack Host Immunity Leading to Cancers	September 23–28	Nairobi, Kenya
2019	IUIS-ALAI-SMI- IMMUNO-INFORMATICS	Immuno-Informatics	April 8–10	Mexico City, Mexico
**Planned courses**:				
2019	IUIS-IDA-SANTHE-FAIS- IMMUNO-SOUTH AFRICA 3	Vaccine Design and Vaccine-induced Immune Responses to HIV, Malaria, and TB	October 7–11	Cape Town, RSA
2019	IUIS-IIS-FIMSA-IMMUNO-INDIA	Basic and Advanced Translational Immunology	October 12–16	Jaipur, India
2019	IUIS-FAIS-IMMUNO-BENIN	Impact of Tropical Infections on Mother and Child Immunity	November 3–10	Ouidah, Benin
2020	IUIS-FAIS-IMMUNO-ETHIOPIA 2	Neglected Tropical Diseases and Malaria Challenges in Sub-Saharan Africa	February 23–29	Bahir Dar, Ethiopia
2020	IUIS-FAIS-IMMUNO-ALGERIA	Allergies and the Immune System	June 24–28	Algiers, Algeria
2020	IUIS-ALAI-IMMUNO-ARGENTINA	Immunological Memory in Infections	tbd	Cordoba, Argentina

The teaching activities of the EDU committee require the voluntary commitment of a dedicated faculty including experts for the various topics from different countries around the globe. Faculty members for IUIS-EDU courses are recruited through the EDU committee but also on the basis of personal interaction of scientists working in closely related fields. We aim to reach a balance between regional and international speakers, and we constantly make efforts to recruit new speakers for our various courses (with a focus on young colleagues). Needless to say that enthusiasm for teaching in an international setting is a “must.” If you are interested to teach in one of the future IUIS-EDU courses, we encourage you to contact us by email.

### Funding of IUIS-EDU Courses

A major issue of the organization of the EDU courses is how to secure sufficient funding. While all IUIS-EDU courses receive some basic funding from the EDU budget, this seed money is never sufficient to cover all expenses. Some courses have received additional funding from the IUIS Clinical Immunology Committee (CIC). Ideally, we aim to provide full support to accepted participants, which would include accommodation, meals, and transport. Local organizers are asked to seek government, University and private sector funding. In addition, members of the EDU Committee undertake substantial efforts to write proposals and recruit additional funds through public and private foundations, industry and other sources. Specifically, the Bill and Melinda Gates Foundation (BMGF), the Volkswagen Foundation (VWF) and the National Institutes of Allergy and Infectious Disease (NIAID), NIH have supported IUIS-EDU courses in Sub-Saharan Africa and Latin America (BMGF: Ethiopia, Brazil;VWF: Gambia, Benin, Ethiopia; NIAID: South Africa).

## Novel On-Line Learning: Immunopaedia

Immunopaedia (https://www.immunopaedia.org.za/) is the IUIS endorsed immunology learning site used for the purpose of IUIS-EDU courses. It was initiated by one of us (CMG), initially for training South African pediatricians in immunology. Immunopaedia has since developed from its inception in 2005. The original aim was to educate pediatricians on the basics of HIV immunology using clinical case studies to highlight an immunological concept. This was termed the “Trojan Horse” approach (3) and over time, Immunopaedia evolved to encompass a broader spectrum of the discipline. However, still at its core is the use of case studies to highlight the immunological defects leading to clinical abnormalities.

From 2015, we began to use Immunopaedia as an on-line pre-course primer. For each of the IUIS-EDU courses, we develop 6–10 learning modules that provide up to date “core” immunology and relevant topic-related teaching material. Core Immunology Modules include (i) a snapshot of the immune system, (ii) ontogeny of the immune system, (iii) the innate immune system, (iv) MHC and antigen presentation, (v) overview of T cell subsets, (vi) thymic T cell development, (vii) B cell activation and plasma cell differentiation, (viii) antibody structure and classes, and (ix) central and peripheral tolerance, and uses specially written material, interviews and plenty of graphics. Specific themed modules are also developed to support the IUIS-EDU course focus areas. These have included: vaccines, immune regulation, pathogens, cancer and autoimmunity, immunotherapy for example. The modules are compiled at least 2–3 months before each course and then made available to students 6 weeks ahead of the specific course. All faculty members are requested to contribute teaching material related to their specific topic. Students on the other hand are requested to prepare themselves for the course by studying the on-line material and scoring >75% on set quizzes at the end of each module. These questions are provided by the faculty, although in some cases the Immunopaedia team also devises questions. After the learner finishes each set of Multiple Choice Questions (MCQs) an automated email of their grade is sent with correct and wrong answers, and why the answer is wrong. In 2018, 100% of the 145 registered participants of the various courses completed the pre-courses MCQs.

Immunopaedia also provides on-line evaluation forms to participants after the completion of an IUIS course. The feedback from the participants is very important as a guideline for further improvement. All course participants have free access to the Immunopaedia content, not only of their specific course, but of all Immunopaedia material indefinitely. The Immunopaedia content provides a rich knowledge resource for all fields of basic and clinical immunology. The offering of a pre-course is based on the “flipped” classroom approach which has been applied to other disciplines (4) and where at least one US tertiary institution has done away with lectures to medical students altogether (5). Such a blended learning approach has proved to be successful for some disciplines (1, 4), but questionable for others (6) and success is most likely dependent on course design. Pre-course on-line games for learning innate immunity, in the context of an accredited university course, has proved successful (2). The long term goal of the IUIS-EDU is to develop pre-course materials with a measurable outcome for the short 5–7 days duration.

A further aim of Immunopaedia has been to build a network of scholars from all IUIS-EDU courses by inviting highly active and motivated students who score exceptionally well on quizzes and questions to become Immunopaedia Ambassadors. Each Ambassador is networked via social media and the objective is to create a sustainable learning environment beyond the end of the course and a network of young immunologists. The tasks of the Ambassadors are to promote Immunopaedia in their hosting institution and to provide content, such as “breaking news” or interviews with prominent immunologists in their region. An example of inputs by Ambassadors were video recordings of prominent immunologists who attended the XII Congress of the Latin American Association of Immunology (ALAI) in 2018 (https://www.immunopaedia.org.za/interviews/video-interviews/alaismi-special-video-interviews/). We currently have 60 Ambassadors spanning Africa, Asia, Europe, North America, Oceania and South America and we feature an ambassador monthly. Each young immunologist has a dedicated space on the site and a link to their own website or other on-line presence. We have also partnered with Virtual Keystone Symposia (https://virtual.keystonesymposia.org/ks/) where Amabassadors can be selected to present their immunology research through Sci-Talks. Through this mechanism, the Ambassador platform provides additional networking and career-boosting opportunities.

## Concluding Remarks

As summarized in this article, the activities of the EDU Committee have considerably expanded in recent years. With the initiative in 2015 to organize 3 to 4 structured IUIS courses per year we have substantially helped to promote the education and training of young scientists in LIC/LMICs. Mentoring at various levels beyond pure science through grant and research project writing, sessions on how to prepare a scientific CV and events promoting women in science is an important asset of IUIS courses. We regard this approach as having a sustainable impact on young scientists, as exemplified by the formation of networks of young immunologists in West Africa, or the recruitment of Immunopaedia “Ambassadors” to promote the unlimited potential of the on-line teaching platform. The ultimate aim of EDU activities is to fulfill the IUIS vision of “Immunology Without Borders.”

## Author Contributions

DK, ML, and CG have equally contributed to this manuscript. All authors agree to be accountable for the content of the work.

### Conflict of Interest Statement

The authors declare that the research was conducted in the absence of any commercial or financial relationships that could be construed as a potential conflict of interest.
