# Supplementary material for: Immunology Education Without Borders
Source: Front Immunol. 2019 Aug 28;10:2012. doi: 10.3389/fimmu.2019.02012 (PMC6724660; doi:10.3389/fimmu.2019.02012)
Supplement: Supplementary file 1 [file Table_1.docx]

**Supplemental Table 1: Current members of the IUIS Education Committee (EDU)**

EFIS^1^: Dieter Kabelitz, Germany (Chair)

Olivier Boyer, France

Angela Santoni, Italy

ALAI^2^: Luis Fernando Garcia, Colombia

Maria Rosa Bono, Chile

Guillermo Docena, Argentina

Rosana Pelayo, Mexico

FIMSA^3^: Shubhada V. Chiplunkar, India

Mehrnaz Mesdaghi, Iran

Bo Huang, China

Jose Alejandro Lopez, Australia

FAIS^4^: Clive Gray, South Africa (Vice-Chair)

Ridha Barbouche, Tunisia

Lucy Ochola, Kenya

North America: Michelle Letarte, Canada (Past-Chair)

Hanne Ostergaard, Canada

Gail Bishop, USA

^1^European Federation of Immunological Societies ^2^Latin American Association of Immunology

^3^Federation of Immunological Societies of Asia-Oceania

^4^Federation of African Immunological Societies
